# Sex Disparities in Long-Term Mortality after Paclitaxel Exposure in Patients with Peripheral Artery Disease: A Nationwide Claims-Based Cohort Study

**DOI:** 10.3390/jcm10132978

**Published:** 2021-07-02

**Authors:** Christian-Alexander Behrendt, Art Sedrakyan, Konstantinos Katsanos, Joakim Nordanstig, Jenny Kuchenbecker, Thea Kreutzburg, Eric A. Secemsky, Eike Sebastian Debus, Ursula Marschall, Frederik Peters

**Affiliations:** 1 Research Group GermanVasc, Department of Vascular Medicine, University Medical Center Hamburg-Eppendorf, 20246 Hamburg, Germany; j.kuchenbecker@uke.de (J.K.); t.kreutzburg@uke.de (T.K.); s.debus@uke.de (E.S.D.); f.peters@uke.de (F.P.); 2 Healthcare Policy and Research, Weill Cornell Medical College, New York, NY 10065, USA; ars2013@med.cornell.edu; 3 Department of Interventional Radiology, Patras University Hospital, 26504 Patras, Greece; katsanos@med.upatras.gr; 4 The Department of Molecular and Clinical Medicine, Institute of Medicine, The Sahlgrenska Academy, University of Gothenburg, 41345 Gothenburg, Sweden; joakim.nordanstig@vgregion.se; 5 Vascular Surgical Department, Sahlgrenska University Hospital, 41345 Gothenburg, Sweden; 6 Beth Israel Deaconess Medical Centre, Division of Cardiology, Smith Center for Outcomes Research in Cardiology, Boston, MA 02215, USA; esecemsk@bidmc.harvard.edu; 7 BARMER, 42266 Wuppertal, Germany; ursula.marschall@barmer.de

**Keywords:** peripheral arterial occlusive disease, chronic limb-threatening ischemia, drug-eluting stent, drug-coated balloon, paclitaxel

## Abstract

Background: Randomized controlled trials have reported excess mortality in patients treated with paclitaxel-coated devices versus uncoated devices, while observational studies have reported the opposite. This study aims to determine the underlying factors and cohort differences that may explain these opposite results, with specific focus on sex differences in treatment and outcomes. Methods: Multicenter health insurance claims data from a large insurance fund, BARMER, were studied. A homogeneous sample of patients with an index of endovascular revascularization for symptomatic peripheral arterial occlusive disease between 2013 and 2017 was included. Adjusted logistic regression and Cox regression models were used to determine the factors predicting allocation to paclitaxel-coated devices and sex-specific 5-year all-cause mortality, respectively. Results: In total, 13,204 patients (54% females, mean age 74 ± 11 years) were followed for a median of 3.5 years. Females were older (77 vs. 71 years), and had less frequent coronary artery disease (23% vs. 33%), dyslipidemia (44% vs. 50%), and diabetes (29% vs. 41%), as well as being less likely to have a history of smoking (10% vs. 15%) compared with males. Mortality differences were mostly attributable to the female subgroup who were revascularized above the knee (hazard ratio, HR 0.78, 95% CI: 0.64–0.95), while no statistically significant differences were observed in males. Conclusions: This study found that females treated above the knee benefited from paclitaxel-coated devices, while no differences were found in males. Ongoing and future registries and trials should take sex disparities into account.

## 1. Introduction

In 2018, a systematic review and meta-analysis of summary-level data from randomized controlled trials (RCT) revealed an association between treatment with paclitaxel-coated devices and increased overall mortality among patients with peripheral arterial occlusive disease (PAOD) [1]. This association was later confirmed in a patient-level meta-analysis and a separate meta-analysis concerning amputation-free survival after below-the-knee treatment [2,3]. During this ongoing controversy, multiple studies that used either real-world data from large administrative and clinical registries [4,5,6,7,8,9] or interim analyses from a large RCT [10] were not able to replicate this unsettling safety signal. Strikingly, analyses using observational datasets found an opposite signal, with improved survival in patients exposed to paclitaxel when compared with those not exposed [5,6]. Ever since, the global scientific community, numerous task forces, and regulatory bodies have discussed the possible factors and cohort differences driving these contrasting results (Supplementary Table S1).

Meanwhile, there is clear evidence of striking differences between the sexes concerning various aspects of PAOD treatments (including prevention, best medical treatment, and revascularization procedures) and outcomes [11,12,13,14,15].

The current study aimed to determine the interaction of sex and corresponding differences in risk profiles on long-term mortality in patients treated with paclitaxel-coated devices, with particular attention given to the role of pharmacological therapy.

A large, unselected, all-comer administrative registry covering more than 13% of the insured cohort in Germany was used for the current study. To minimize the risk of bias caused by heterogeneity among the study groups, we tailored the cohort to a sample of homogenous patients with first endovascular interventions.

## 2. Materials and Methods

### 2.1. BARMER Cohort

The longitudinal data of Germany’s second-largest insurance fund, BARMER, include the outpatient and inpatient medical care provided to approximately 9.4 million German citizens (13.2% of Germany’s insured population), involving 9.5 million hospitalizations between 2013 and 2017. Furthermore, comprehensive information on pharmacological treatments is available in the same database. The BARMER cohort includes nationally generalizable data with comparable sex and age distributions to the entire German population and has been widely used for cardiovascular research [16]. The database contains longitudinal information for each person, including date of birth, start and end of insurance episodes, and date of death, through to 31 December 2019. A regular random sample validation of internal and external validity was performed by the Medical Service of the Health Funds (MDK) in Germany, and various validation studies have been published [17,18,19,20].

We used the International Classification of Diseases in its German Modification (ICD-10-GM) to identify diagnoses and the Operations and Procedures Codes (OPS) coding to identify procedures. The German OPS code is adapted to the International Classification of Procedures in Medicine (ICPM). For identifying medical prescriptions, the German version of the international Anatomical Therapeutic Chemical (ATC) classification was used. The study protocol was published a priori on 24 December 2020 (clinicaltrials.gov NCT04683458) (accessed on 1 June 2021) [21].

### 2.2. Study Population

We included patients with a primary diagnosis of intermittent claudication (IC) (I70.22 until 2014 and I70.21-22 since 2015), chronic limb-threatening ischemia (CLTI) (I70.22-24 until 2014 and I70.23-25 since 2015), or IC and CLTI as secondary diagnosis, in combination with a primary diagnosis of diabetic foot syndrome (E10.50-51, E10.7, E11.50-51, E11.7), other peripheral vascular diseases (I73), arterial embolism and thrombosis (I74), cellulitis of the fingers and toes including acute lymphangitis (L03.01-02, L03.11), or chronic ulcers of the skin and gangrene (L98.4, R02) using the ICD-10-GM (Supplementary Table S2).

The index admission for symptomatic PAOD, denoted as “index stay”, was identified between 1 January 2005 and 31 December 2017, with follow-up through 31 December 2019.

Exclusion criteria were an index stay before 2013, an age below 40, hybrid surgery, revascularization at other levels outside of the femoropopliteal or crural arteries, previous endovascular intervention, surgical revascularization, coronary angioplasty, major amputation of the lower limbs, or any other exposure to paclitaxel or cancer diagnoses during the five years before the index procedure. We also excluded patients who were not continuously insured at BARMER during the 5 years before the index stay. These selection criteria were aimed at tailoring the study to a cohort as homogenous as possible with respect to prior diagnoses and interventions, but also to prior paclitaxel exposure due to coronary intervention or cancer treatment. There were a few cases with missing information on age, sex, and follow-up (~0.5%), and these were excluded.

Patients who received at least one index drug-coated balloon/stent at the index stay were assigned to the paclitaxel group. If the patient received a stent and a balloon at the same time, we defined it as a stent procedure. Additional information and coding criteria for drug-eluting stent or drug-coating balloon can be found in Supplementary Table S2.

### 2.3. Baseline Characteristics

Primary and secondary diagnoses reported during the index stay or during inpatient admissions up to five years prior to the index stay (the lookback period) were used to measure comorbidities, including coronary artery disease, dyslipidemia, frailty, a history of myocardial infarction, a history of stroke or transient ischemic attack (TIA), and the Elixhauser comorbidity groups (i.e., congestive heart failure, cardiac arrhythmias, hypertension, neurodegenerative disorders, chronic pulmonary disease, uncomplicated diabetes, complicated diabetes, hypothyroidism, obesity, weight loss, and depression) [22,23]. The linear van Walraven score (vWS), a weighted sum score, ranged from −19 to +89 based on the Elixhauser groups (wherein high scores represent a higher risk for in-hospital mortality) was also calculated [22,23]. We evaluated the validity of these comorbidities over time thoroughly in an earlier study [24]. Smoking was defined as ICD-10-GM code F17: either noted during an outpatient visit one year prior to the index stay or during an inpatient visit within five years of the index stay. Further, we measured the number of inpatient visits, PAOD-related outpatient visits, and optimal pharmacological treatment (consisting of lipid-lowering, antithrombotic, and antihypertensive drugs) [25], oral anticoagulation, and the number of different prescriptions during the year before the index stay. At the index stay, we ascertained age, hospital volume (lower or higher than median), the number of invasive revascularizations at index, the length of hospital stay, IC vs. CLTI at presentation, discharge year (2013–14 vs. 2015–17), patient residence (West or East Germany), whether a stent was placed (vs. a balloon angioplasty), and whether below-the-knee arteries were involved (vs. above-the-knee arteries).

### 2.4. Statistical Analysis

The baseline characteristics are presented as proportions for categorial variables, means (with standard deviation) for normally distributed variables, and medians (with interquartile ranges) for non-normally distributed variables. We computed standardized mean differences (SMD), where values greater than or equal to 0.1 denote meaningful differences between males and females. In observational studies, which usually involve large numbers of participants, the use of SMD instead of *p*-values is highly recommended to avoid false-positive findings.

Logistic regression was applied for modelling the relation between the baseline variables and the odds of receiving a paclitaxel-coated device versus receiving a non-coated device during the index procedure. This was expressed as an odds ratio with 95% confidence intervals. The top five predictors were identified using the variable importance metric suggested by Breiman [26].

The primary outcome was a 5-year, all-cause mortality with the end of the follow-up in December 2019. Follow-up times longer than five years were censored to ensure robust estimations. There was no exclusion of patients with a shorter follow-up. Cox proportional hazard regression models were utilized to estimate the impact of paclitaxel exposure mortality for the total cohort and the subgroups of paclitaxel-coated device type (balloon vs. stent), affected level (above vs. below the knee), hospital volume (low vs. high), patient residence (West vs. East Germany), Fontaine stage (CLTI vs. IC), history of diabetes (no vs. yes), van Walraven score (<5 vs. ≥5), prior PAOD diagnosis (no vs. yes), and history of coronary artery disease (no vs. yes). Each model was estimated separately for the total cohort and each subgroup was adjusted for all baseline variables, resulting in point estimates of the hazard ratio (HR) of the impact of paclitaxel exposure on 5-year, all-cause mortality with 95% confidence intervals. An interaction of each binary subgroup variable and binary paclitaxel exposure was entered into each model to compute separate confidence intervals for both subgroups. If the confidence intervals of females and males were non-overlapping, we tested sex differences using the three-way interactions of sex, paclitaxel, and the variable of interest. The proportional hazards assumption was checked using graphical diagnostics based on Schoenfeld residuals, and the test suggested by Grambsch and Therneau [27]. This is an explanatory analysis not adjusting for multiple testing.

A landmark analysis (removing all deaths up to one year after the index stay) was applied for assessing the role of optimal pharmacological treatment (OPT) during the year after discharge from the index stay (Supplementary Table S3).

Data management was performed with the software SAS, version 9.04 (SAS Institute, NC, USA). We reported results using the reporting of studies conducted using the observational routinely-collected health data (RECORD) statement [28] and the Strengthening the Reporting of Observational studies in Epidemiology (STROBE) statement [29].

The statistical analyses and visualizations were performed with software R version 3.6.2 (The R Foundation for Statistical Computing, Vienna, Austria). Illustrations were designed using Adobe Illustrator version 24.0.1 (Adobe Systems Software Ireland Ltd., Dublin, Republic of Ireland).

## 3. Results

**Figure 1:**
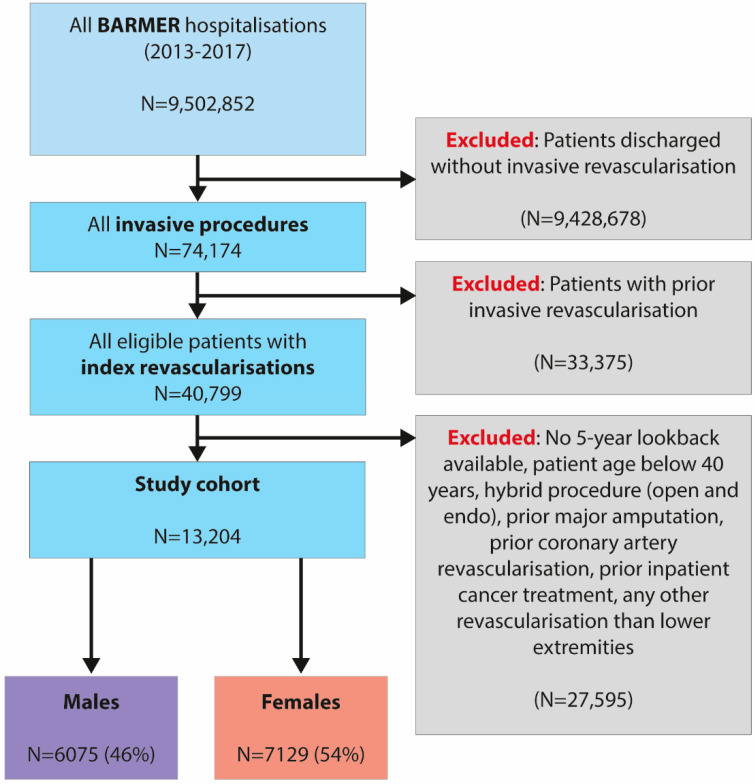
Flow chart of the current analysis of health insurance claims data from Germany.

A total of 13,204 patients (54% females, mean age 74.4 ± 10.7 years) met the inclusion criteria between 2013 and 2017. Female and male patients were followed for a median of 1274 (IQR 846-1798) and 1302 (IQR 874-1817) days, respectively (Figure 1 and Table 1).

### 3.1. Baseline Characteristics by Sex

The baseline characteristics of the entire study cohort by sex with standardized mean differences (SMD) are presented in Table 1. While females were selected for first endovascular interventions at a higher age (77 vs. 71 years, SMD = 0.549), they exhibited a favorable cardiovascular risk profile in terms of coronary artery disease (23% vs. 33%, SMD = 0.241), dyslipidemia (44% vs. 50%, SMD = 0.110), diabetes (29% vs. 41%, SMD = 0.255), and smoking (10% vs. 15%, SMD = 0.149) when compared with their male counterparts. Females were more often diagnosed with depression (10% vs. 6%, SMD = 0.142) and hypothyroidism (22% vs. 8%, SMD = 0.391) relative to males.

During the year before the index admission, females had less often experienced an outpatient visit for PAOD (55% vs. 60%, SMD = 0.112) and were less often treated with optimal pharmacological treatment (19% vs. 27%, SMD = 0.190), but had an overall higher mean number of different pharmacological prescriptions (10 vs. 9, SMD = 0.144).

The five strongest predictors increasing the odds of being treated with paclitaxel-coated devices in females were a discharge year later than 2014, high center volume, intermittent claudication, and uncomplicated diabetes, while a higher van Walraven score decreased the odds accordingly. In males, a discharge year later than 2014, high center volume, intermittent claudication, and residency in East Germany were associated with higher odds of being treated with paclitaxel-coated devices, while older age decreased the odds accordingly (Supplementary Table S4).

### 3.2. Impact of Paclitaxel Exposure on 5-Year Mortality among Subgroups

**Figure 2:**
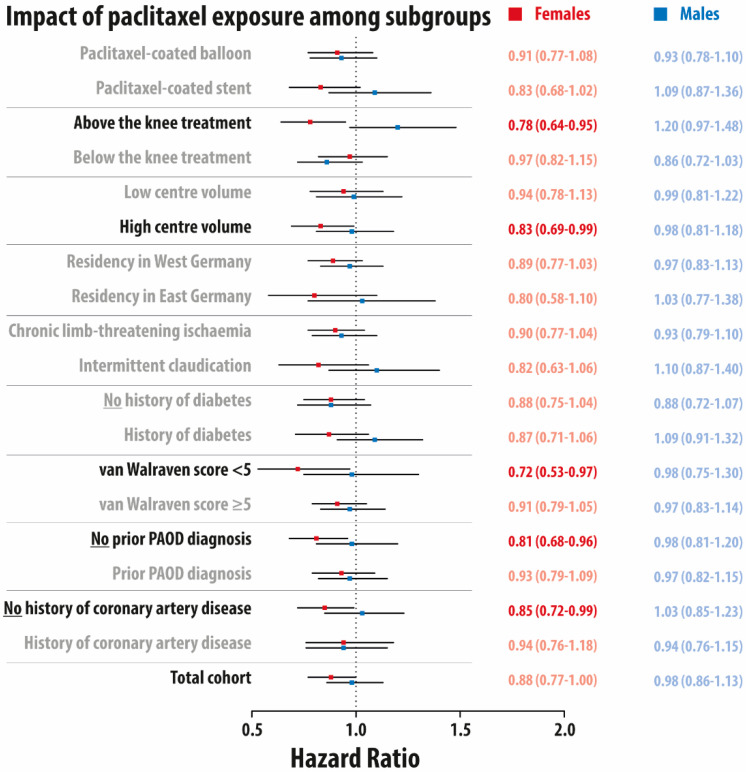
Forest plot for the adjusted impact of paclitaxel exposure on 5-year overall mortality among different subgroups stratified by sex (full cohort). Hazard ratio and 95% confidence interval in red for females and in blue for males. PAOD: peripheral arterial occlusive disease. All models were adjusted for all baseline characteristics using Cox models with regression adjustment.

The sex-stratified impact of paclitaxel exposure in the total cohort and different subgroups using variables available until index admission is presented in Figure 2. No statistically significant effect was apparent in males. In females, paclitaxel exposure was associated with a lower mortality in the following subgroups: revascularization of lesions above the knee (HR 0.78, 95% CI: 0.64–0.95), higher center volume (HR 0.83, 95% CI: 0.69–0.99), lower van Walraven score < 5 (HR 0.72, 95% CI: 0.53–0.97), no prior history of PAOD during the outpatient course (HR 0.81, 95% CI: 0.68–0.96), and no history of coronary artery disease (HR 0.85, 95% CI: 0.72–0.99). No statistically significant effect was apparent in males.

### 3.3. Interaction of Treatment Level, Sex, and Paclitaxel Exposure on 5-Year Mortality

**Figure 3:**
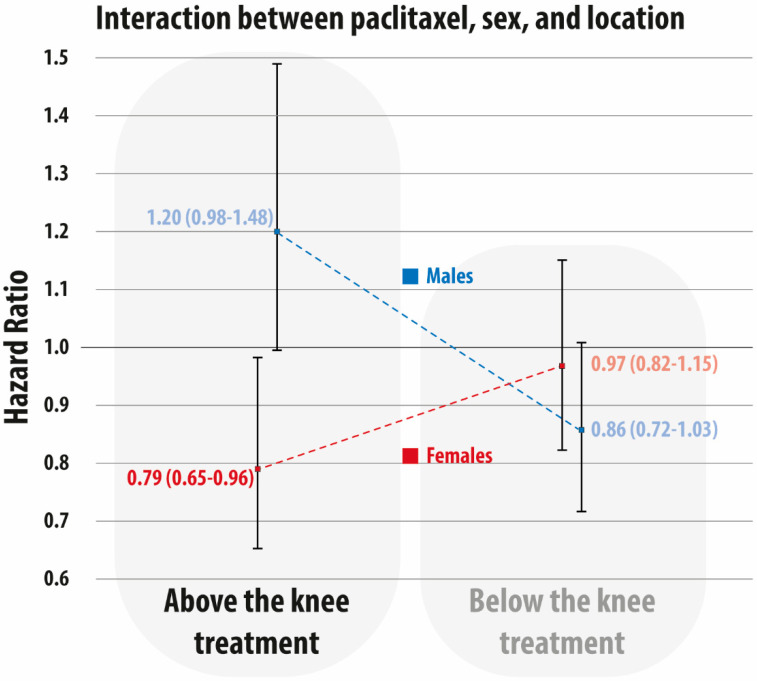
Interaction between paclitaxel exposure (yes vs. no), sex (female vs. male), and level (above-the-knee treatment vs. below-the-knee treatment). Hazard ratio and 95% confidence interval are in red for females and in blue for males.

The interaction between treatment level (above the knee vs. below the knee), dichotomized sex, and paclitaxel exposure on the 5-year mortality hazard ratio are presented in Figure 3. While no statistically significant differences were observed in the subgroup treated below the knee, a significantly lower 5-year mortality was observed in females (HR 0.79, 95% CI: 0.65–0.96) when compared with males (HR 1.20, 95% CI: 0.98–1.48) (*p*-value for interaction between males and females = 0.003) in the subgroup treated above the knee.

## 4. Discussion

This analysis of unselected all-comer administrative data from Germany aimed to further determine the underlying factors that were driving the observed improvement in mortality associated with the use of paclitaxel-coated devices. Our design, focusing on first endovascular interventions in a cohort which was as homogenous as possible, revealed that the mortality differences were mostly attributable to the female subgroup treated above the knee, while no statistically significant differences were observed in males.

These novel findings emphasize the results from a recent patient-level meta-analysis of a drug-coated balloon [30]. Indeed, it appears difficult to imagine how the application of an endovascular device could directly improve or worsen the long-term outcomes years after the index treatment. Hence, paclitaxel exposure may instead serve as proxy for the underlying confounders related to the post-discharge surveillance. That said, the interesting fact that females with no prior history of PAOD were especially positively impacted by paclitaxel exposure further generates hypotheses related to post-discharge management. If females were systematically underdiagnosed and undertreated before their first hospital admission, they would likely benefit from an evidence-based surveillance strategy including all aspects of best medical treatment after discharge. Interestingly, these differences occurred even though we made all attempts to minimize the influence of interventions occurring before the index stay or differences in treatment at the index. For instance, prior coronary interventions and related adverse events, but also optimal pharmacological therapy, were more common among males than females before excluding patients with hybrid surgery, prior cancer diagnosis, and prior coronary or peripheral interventions. Consequently, the observed survival benefit associated with paclitaxel exposure in females might be a sign of improved management in a subgroup of patients who exhibited insufficient prior care. This explanation would be in line with the well-known concept in health behavior research called the “teachable moment of the first in-hospital intervention”.

The surveillance and patients’ compliance in following pharmacological prescriptions beyond discharge was likely different between the sexes. A striking underrepresentation of females in RCT and varying outcomes between sexes were reported before the paclitaxel controversy was initiated in December 2018 [15,31,32,33,34,35]. Interestingly, neither RCT nor real-world studies during the ongoing paclitaxel controversy studied a possible interaction of sex and lesion level with outcomes after paclitaxel exposure. While females treated above the knee benefited from paclitaxel exposure, no statistically significant differences were seen if procedures were applied below the knee. This may indicate that these two groups are fundamentally different. Although the clinical symptoms had no impact, a comparable trend was observed in the subgroup of patients with intermittent claudication. In sum, it appears likely that females selected in an earlier disease stage will benefit most from secondary prevention. Although the proportion of females may not have an impact on central conclusions in appropriately powered RCT, the vulnerable aspects of trial design and power calculation in subgroups may still be affected by underrepresented subgroups. Even the most recent RCTs are likely affected by relevant bias. For instance, in a subgroup analysis of the VOYAGER PAD trial, the observed benefit of the investigational treatment on the primary efficacy endpoint was only driven by the male subgroup (HR 0.82, 95% CI: 0.71–0.94) while no differences were observed in females (HR 0.97, 95% CI: 0.76–1.23) [36]. Interestingly, the authors recently presented a subgroup analysis of that trial concluding that there was no mortality difference in patients exposed to paclitaxel-coated devices vs. those not exposed.

While 28 RCTs in the first meta-analysis enrolled 33% females [1] and 8 RCT in the second meta-analysis enrolled 29% females [2], the current study was undertaken within a dataset more representative of everyday clinical practice that included 54% females. The latter seems more representative for the disease under study since a recent comparison of more than 1 million hospitalizations in 11 countries revealed that females represent approximately 40% (between 23% in Portugal and 46% in Sweden) of the cohort treated with both open-surgical and endovascular revascularizations [11]. A recent systematic review of 69 PAOD trials in the USA showed that females were appropriately represented in less than 16% of these trials, while the percentage of females in the underlying PAOD population was 53.1% [34].

Regulatory bodies and societies issued still-existing safety warnings concerning the use of paclitaxel-coated devices for the treatment of PAOD [37]. Interventionists, patients, and the medical device industry face a huge challenge to interpret an evidence base which is hardly comprehensible or understandable [38]. The current study from the Medical Device Epidemiology Network (MDEpiNet) aims to support the ongoing discussion and regulatory decision-making.

A particular merit of our study is the rigorous design focussing on first endovascular intervention and excluding patients with either prior interventions or a potential paclitaxel exposure due to coronary intervention or cancer treatment. Mimicking an intention-to-treat approach known in pharmacoepidemiology, this enabled the study to avoid distortions due to prevalent user bias and immortal time bias, caused by including patients with reinterventions or at different stages of severity of atherosclerotic disease progression. The current discussion and the careful analysis of underlying real-world data can probably serve as an example to illustrate two central conclusions. First, there was no evidence for excess mortality in any of the subgroups studied. Moreover, females revascularized above the knee along with an optimal pharmacological post-discharge treatment likely benefitted from being exposed to paclitaxel-coated devices. Second, while the community is currently discussing marginal differences concerning primary efficacy endpoints in recently completed trials on new medical devices and cardiovascular protective drugs, we tend to neglect that an evidence-based and cost-effective basic optimal pharmacological treatment could significantly improve outcomes by 30% [12,39,40]. Furthermore, the complementary interaction between all therapeutic modalities including best medical treatment, invasive revascularization, and supervised exercise therapy, deserves a more thorough consideration [41].

This study had limitations. First, there is an ongoing discussion concerning the comprehensive value of administrative registries for research. Indeed, health insurance claims are primarily collected for the reimbursement and administration of medical care. However, they are universally used to monitor healthcare services and quality improvement [16]. There is growing data of their good internal and external validity, especially regarding outcomes with major health impacts [42]. Nevertheless, a selection bias with possible impacts on the intervention-outcome relationship cannot be ruled out by design (e.g., through excluded patients) and by the fact that currently, no health insurance fund in Germany can claim to cover the entire insured population. Second, the granularity of the study data limits its use. No information was available on anatomical details or lesion severity. Furthermore, the specific devices were not collected. Collaborations such as the Medical Device Epidemiology Network (www.mdepinet.net, accessed on 1 June 2021) can help to develop pragmatic ways to collect device identifiers in routinely collected data. Lastly, even though the current study used robust statistical methods and numerous confirmative sensitivity analyses, the issue of residual confounding in observational research remains unsolved to date. Therefore, due to the non-random assignment, the current study can only generate hypotheses and reveal associations. Yet, appropriately powered and independent RCTs are still not available to further determine causal exposure–outcome relationships [43].

## 5. Conclusions

This study found that females treated above the knee benefit from paclitaxel-coated devices, while no differences were found in males. Ongoing and future registries and trials should take sex disparities into account.

## Figures and Tables

**Table 1:** Baseline characteristics by female vs. male sex in this retrospective analysis of health insurance claims data from Germany.

	*N*	% Females	*N*	% Males	SMD
No of patients	7129	100	6075	100	
Paclitaxel exposure at index	1611	22.6	1324	21.8	0.017
Stent at index	3030	42.5	2643	43.5	0.020
Crural arteries involved	2509	35.2	2254	37.1	0.038
Intermittent claudication	3949	55.4	3639	59.9	0.091
Discharge year >2014	4434	62.2	3791	62.4	0.005
High hospital volume	3736	52.4	3177	52.3	0.001
Patient residence East Germany	1355	19.0	1361	22.4	0.086
Prior outpatient PAOD visit	3907	54.8	3663	60.3	0.112 #
Van Walraven score >5	3593	50.4	2855	47.0	0.069
Coronary artery disease	1611	22.6	2023	33.3	0.241 #
Dyslipidemia	3151	44.2	3019	49.7	0.110 #
History of myocardial infarction	364	5.1	377	6.2	0.046
History of stroke or TIA	549	7.7	504	8.3	0.021
Congestive heart failure	1576	22.1	1318	21.7	0.011
Cardiac arrhythmias	1739	24.4	1567	25.8	0.034
Hypertension	6003	84.2	4878	80.3	0.102 #
Neurodegenerative disorders	428	6.0	413	6.8	0.033
Chronic pulmonary disease	984	13.8	778	12.8	0.030
Diabetes, uncomplicated	1668	23.4	1895	31.2	0.177 #
Diabetes, complicated	1112	15.6	1458	24.0	0.214 #
Diabetes, total	2082	29.2	2509	41.3	0.255 #
Hypothyroidism	1547	21.7	486	8.0	0.391 #
Obesity	763	10.7	796	13.1	0.074
Weight loss	349	4.9	164	2.7	0.115 #
Depression	713	10.0	377	6.2	0.142 #
Smoking	741	10.4	936	15.4	0.149 #
Optimal pharmacological therapy during the prior year	1355	19.0	1640	27.0	0.190 #
Oral anticoagulation during the prior year	1119	15.7	1027	16.9	0.030
Age, mean (SD)	N/A	77.01 (10.15)	N/A	71.34 (10.51)	0.549 #
Prior hospital visits, mean (SD)	N/A	0.76 (1.21)	N/A	0.76 (1.27)	<0.001
No of different prescriptions during the prior year, mean (SD)	N/A	10.03 (5.75)	N/A	9.19 (5.85)	0.144 #
Number of surgeries at index, mean (SD)	N/A	1.76 (1.41)	N/A	1.78 (1.69)	0.007
Hospital length of stay, mean (SD)	N/A	5.85 (8.48)	N/A	5.54 (9.01)	0.036
Follow-up time, median [Q1, Q3]	N/A	1274 [846.0, 1798.0]	N/A	1302 [874.0, 1816.5]	0.047

Footnote: PAOD = peripheral arterial occlusive disease; SMD = standardized mean differences; TIA = transient ischemic attack; SD = standard deviation; N/A = not applicable. # denotes meaningful differences.

## Data Availability

The data presented in this study are available on request from the corresponding author. The data are not publicly available due to legal restrictions.

## Supplementary Materials

The following are available online at https://www.mdpi.com/article/10.3390/jcm10132978/s1: Table S1: Baseline characteristics of randomised controlled trials included in two systematic reviews in 2018 and 2020 vs. the current study; Table S2: International classification of diseases (ICD) 10th revision, operational and procedure coding (OPS), and anatomical-therapeutical-chemical (ATC) classification used for this study; Table S3: Top five strongest predictors increasing or decreasing the odds of being treated with paclitaxel coated devices; Table S4: Baseline characteristics by male vs. female sex of landmark sample.
