# Supplementary material for: Sex Disparities in Long-Term Mortality after Paclitaxel Exposure in Patients with Peripheral Artery Disease: A Nationwide Claims-Based Cohort Study
Source: J Clin Med. 2021 Jul 2;10(13):2978. doi: 10.3390/jcm10132978 (PMC8268810; doi:10.3390/jcm10132978)
Supplement: Supplementary file 1 [file jcm-10-02978-s001.zip › jcm-1271413-supplementary.pdf]

Supplemental Material

Supplemental Table S1: Baseline characteristics of randomised controlled trials included in two systematic reviews in 2018 and 2020 vs. the current study.

Name	N	Age (y)	Male	Smoking	Diabetes	IC
Above the knee						
ZILVER PTX	479	68	64%	84%	45%	90%
THUNDER	102	68	64%	23%	48%	80%
INPACT SFA	331	68	66%	38%	43%	95%
FEMPAC	87	69	60%	41%	47%	94%
LEVANT I	101	69	63%	35%	48%	93%
LEVANT II	476	68	63%	35%	43%	92%
ILLUMENATE EU	294	68	71%	88%	37%	98%
CONSEQUENT	153	68	68%	48%	37%	100%
ISAR-STATH	100	69	70%	70%	25%	93%
IN.PACT SFA JAPAN	100	73	76%	28%	96%	96%
DEBELLUM	50	67	74%	62%	44%	90%
PACIFIER	91	71	62%	54%	35%	96%
ISAR-PEBIS	70	69	69%	64%	34%	97%
FAIR	119	68	69%	32%	38%	92%
BIOLUX P-I	60	71	57%	68%	33%	83%
RANGER-SFA	105	68	72%	44%	38%	97%
ACOART I	200	66	74%	31%	56%	63%
DEBATE IN SFA	104	75	69%	52%	74%	26%
ILLUMENATE PIVOTAL	300	69	59%	81%	50%	96%
LUTONIX JAPAN	109	74	83%	72%	77%	99%
RAPID	160	68	64%	49%	29%	83%
EFFPAC	171	68	65%	N/A	39%	98%
FINN-PTX	41	68	71%	37%	37%	83%
BATTLE	171	70	73%	28%	37%	81%
DEBATE-SFA	255	73	67%	25%	55%	88%
PACUBA	74	68	58%	47%	41%	99%
DRECOREST	57	68	56%	54%	49%	54%
FREEWAY	204	65	77%	85%	26%	95%
**Weighted mean**		**69**	**67%**	**54%**	**45%**	**89%**
Below the knee						
**The current study**		**74**	**46%**	**23%**	**35%**	**58%**
DEBATE BTK	132	75	80%	15%	100%	0%
INPACT DEEP	358	73	74%	15%	73%	0%
BIOLUX P-II	72	72	79%	14%	67%	22%
ACOART II	120	71	60%	27%	73%	1%
ACOART BTK	85	77	73%	N/A	95%	0%
SINGA-PACLI	138	69	67%	36%	94%	0%
LUTONIX BTK	442	73	69%	14%	70%	10%
HADDAD ET AL	93	N/A	N/A	73%	96%	0%
**Weighted mean**		**73**	**71%**	**22%**	**79%**	**4%**
**The current study**		**74**	**46%**	**23%**	**35%**	**58%**

Footnote: IC = Intermittent Claudication.

Supplemental Table S2: International classification of diseases (ICD) 10th revision, operational and procedure coding (OPS), and anatomical-therapeutical-chemical (ATC) classification used for this study.

Variable	ICD code (or OPS or ATC if indicated)
Symptomatic peripheral arterial occlusive disease	**<2015**:<br>I70.21 Pelvic-leg arteries with exercise induced pain, walking distance < 200m, **Fontaine stage II**<br>I70.22 Pelvic-leg arteries with rest pain, **Fontaine stage III**<br>I70.23-24 Pelvic-leg arteries with ulcerations and/or gangrene, **Fontaine stage IV**<br>**≥ 2015**:<br>I70.21-22 Pelvic-leg arteries with exercise induced pain, **Fontaine stage II**<br>I70.23 Pelvic-leg arteries with rest pain, **Fontaine stage III**<br>I70.24-25 Pelvic-leg arteries with ulcerations and/or gangrene, **Fontaine stage IV**<br>**Others**:<br>E10.50-51 Type 1 diabetes mellitus with peripheral vascular complications<br>E10.7 Type 1 diabetes mellitus with diabetic foot syndrome<br>E11.50-51 Type 2 diabetes mellitus with peripheral vascular complications<br>E11.7 Type 2 diabetes mellitus with diabetic foot syndrome<br>I73.0 Other peripheral vascular diseases, Raynaud syndrome<br>I73.1 Other peripheral vascular diseases, Thrombangiitis obliterans<br>I73.8 Other peripheral vascular diseases<br>I73.9 Other peripheral vascular diseases<br>I74.0 Arterial embolism and thrombosis, aorta abdominalis<br>I74.1 Arterial embolism and thrombosis, aorta<br>I74.2 Arterial embolism and thrombosis, upper extremities<br>I74.3 Arterial embolism and thrombosis, lower extremities<br>I74.4 Arterial embolism and thrombosis, arteries of the extremities<br>I74.5 Arterial embolism and thrombosis, aorta iliacal<br>I74.8 Arterial embolism and thrombosis, other arteries<br>I74.9 Arterial embolism and thrombosis, other arteries<br>L03.01-2, L03.11 Cellulitis of finger and toe including acute lymphangitis<br>L98.4 Chronic ulcer of skin, not elsewhere classified<br>R02 Gangrene, not elsewhere classified
**Interventions**	
Amputation	OPS 5-864 Major amputation, above the ankle<br>5-865 Minor amputation, below the ankle
Percutaneous coronary intervention	8-837, 5-38a.c
Peripheral vascular intervention	8-836, 8-840, 8-841, 8-842, 8-843, 8-844, 8-845, 8-846, 8-847, 8-848, 8-849, 8-83c, 8-84a
Open surgical revascularization	5-380, 5-381, 5-382, 5-383, 5-384, 5-38c, 5-38d, 5-38e, 5-38f, 5-393, 5-394, 5-395, 5-396, 5-98a, 5-38a.4, 5-38a.c
Drug-coated balloon	8-83b.b0, 8-83b.b2-b5, 8-83b.ba-bd
Uncoated balloon	8-836.04/09/0b/0c/0e
Drug-eluting stent (paclitaxel)	8-840.04/9/q/b/s/c/e, 8-841.04/9/q/b/s/c/e, 8-842.04/9/q/b/s/c/e, 8-848.04/9/q/b/s/c/e, 8-83b.03-06
Drug-eluting stent (other than paclitaxel)	8-83b.0a-f, 8-83b.0x
Bare metal stent	8-840.04/9/q/b/s/c/e, 8-840.14/9/q/b/s/c/e, 8-840.24/9/q/b/s/c/e, 8-840.34/9/q/b/s/c/e, 8-840.34/9/q/b/s/c/e, 8-840.44/9/q/b/s/c/e, 8-840.54/9/q/b/s/c/e,
**Medications**	
Optimal pharmacological treatment	Lipid-Lowering drugs & antithrombotics & antihypertensives
Lipid lowering drugs	ATC C10
Antithrombotics	B01
Antihypertensives	C02, C03, C07, C08, C09
Oral anticoagulation	B01AA, B01AE, B01AF
**Covariates**	
Stroke or transient ischaemic attack	I61, I63, I64, G45
Dyslipidaemia	E78
Coronary artery disease	I20-25
Smoking	F17
Myocardial infarction	I20.0, I21-I24
Frailty	A04, A09, A41, B95, B96, D64, E05, E16, E53, E55, E83, E87, F00, F01, F03, F05, F10, F32, G20, G30, G31, G40, G45, G81, H54, H91, I63, I67, I69, I95, J18, J22, J69, K26, K52, K59, K92, L03, L08, L89, L97, M15, M19, M25, M41, M48, M79, M80, M81, N17, N18, N19, N20, N28, N39, R00, R02, R11, R13, R26, R29, R31, R32, R33, R40, R41, R44, R45, R47, R50, R54, R55, R56, R63, R69, R79, R94, S00, S01, S06, S09, S22, S32, S42, S51, S72, S80, T83, U80, X59, Y84, Z22, Z50, Z60, Z73, Z74, Z75, Z87, Z91, Z93, Z99

Footnote: TIA = Transient Ischaemic Attack.

Supplemental Table S3: Top five strongest predictors increasing or decreasing the odds of being treated with paclitaxel coated devices.

	Females			Males	
	**OR**	**95% confidence interval**		**OR**	**95% confidence interval**
**Discharge Year later than 2014**	2.59	(2.27-2.95)	**Discharge Year later than 2014**	2.28	(1.98-2.62)
**High Centre Volume**	1.21	(1.08-1.36)	**High Centre Volume**	1.33	(1.18-1.51)
**Intermittent Claudication**	1.19	(1.03-1.38)	**Intermittent Claudication**	1.14	(1.00-1.30)
**Residency in East Germany**	1.16	(1.00-1.35)	**Diabetes, uncomplicated**	1.23	(1.06-1.42)
**Higher Age**	0.99	(0.97-0.99)	**Higher van Walraven score**	0.99	(0.98-0.99)

Footnote: OR = Odds Ratio.

Supplemental Table S4: Baseline characteristics by male vs. female sex of landmark sample.

	N	% Females	N	% Males	SMD
**No of patients**	**6339**	**100**	**5493**	**100**	
Paclitaxel exposure at index	1464	23.1	1225	22.3	0.021
Stent at index	2732	43.1	2444	44.5	0.028
Crural arteries involved	2054	32.4	1873	34.1	0.036
Intermittent claudication	3841	60.6	3527	64.2	0.074
Discharge year >2014	4434	62.2	3791	62.4	0.005
High hospital volume	3736	52.4	3177	52.3	0.001
Patient residence East Germany	1355	19.0	1361	22.4	0.086
Prior outpatient PAOD visit	3907	54.8	3663	60.3	0.112#
Outpatient PAOD visit after index (1 year)	5990	94.5	5251	95.6	0.052
Van Walraven score >5	2859	45.1	2335	42.5	0.054
Coronary artery disease	1350	21.3	1747	31.8	0.24
Dyslipidaemia	2865	45.2	2741	49.9	0.095
History of myocardial infarction	292	4.6	313	5.7	0.049
History of stroke or TIA	431	6.8	406	7.4	0.025
Congestive heart failure	1173	18.5	1005	18.3	0.004
Cardiac arrhythmias	1344	21.2	1236	22.5	0.032
Hypertension	5293	83.5	4367	79.5	0.104
Neurodegenerative disorders	323	5.1	324	5.9	0.034
Chronic pulmonary disease	837	13.2	648	11.8	0.042
Diabetes, uncomplicated	1407	22.2	1659	30.2	0.182
Diabetes, complicated	906	14.3	1241	22.6	0.215
Diabetes, total	1775	28	2208	40.2	0.259
Hypothyroidism	1376	21.7	412	7.5	0.411
Obesity	697	11	720	13.1	0.065
Weight loss	241	3.8	110	2	0.106
Depression	590	9.3	313	5.7	0.138
Smoking	1287	20.3	1566	28.5	0.194
Optimal pharmacological therapy during the prior year	1192	18.8	1467	26.7	0.189
Optimal pharmacological therapy after index (1 year)	3068	48.4	3016	54.9	0.129
Oral anticoagulation during the prior year	887	14	829	15.1	0.032
Age, mean (SD)		76.06 (9.95)		70.44 (10.16)	0.559
Prior hospital visits, mean (SD)		0.68 (1.12)		0.68 (1.15)	<0.001
No of different prescriptions during the prior year, mean (SD)		9.62 (5.48)		8.77 (5.57)	0.154
Number of surgeries at index, mean (SD)		1.64 (1.22)		1.63 (1.33)	0.012
Hospital length of stay, mean (SD)		5.19 (7.80)		4.88 (8.32)	0.04
Follow-up time, median [Q1, Q3]		1376.00 [981.00, 1826.00]		1391.00 [998.00, 1826.00]	0.02

Footnote: PAOD = Peripheral arterial occlusive disease. SMD = Standardized mean differences.

TIA = Transient ischaemic attack. SD = Standard deviation. # denotes meaningful differences.
